# Drug resistance-conferring mutations in *Mycobacterium tuberculosis* from Madang, Papua New Guinea

**DOI:** 10.1186/1471-2180-12-191

**Published:** 2012-09-04

**Authors:** Marie Ballif, Paul Harino, Serej Ley, Mireia Coscolla, Stefan Niemann, Robyn Carter, Christopher Coulter, Sonia Borrell, Peter Siba, Suparat Phuanukoonnon, Sebastien Gagneux, Hans-Peter Beck

**Affiliations:** 1 Swiss Tropical and Public Health Institute, Socinstrasse 57, 4002, Basel, Switzerland; 2 University of Basel, Basel, Switzerland; 3 Papua New Guinea Institute of Medical Research, Goroka and Madang, Papua New Guinea; 4 Borstel Research Center, Borstel, Germany; 5 Queensland Mycobacterium Reference Laboratory, Pathology Queensland, Brisbane, Australia

**Keywords:** *Mycobacterium tuberculosis*, Papua New Guinea, Drug resistance, Mutations

## Abstract

**Background:**

Monitoring drug resistance in *Mycobacterium tuberculosis* is essential to curb the spread of tuberculosis (TB). Unfortunately, drug susceptibility testing is currently not available in Papua New Guinea (PNG) and that impairs TB control in this country. We report for the first time *M. tuberculosis* mutations associated with resistance to first and second-line anti-TB drugs in Madang, PNG. A molecular cluster analysis was performed to identify *M. tuberculosis* transmission in that region.

**Results:**

Phenotypic drug susceptibility tests showed 15.7% resistance to at least one drug and 5.2% multidrug resistant (MDR) TB. Rifampicin resistant strains had the *rpoB* mutations D516F, D516Y or S531L; Isoniazid resistant strains had the mutations *katG* S315T or *inhA* promoter C15T; Streptomycin resistant strains had the mutations *rpsL* K43R, K88Q, K88R), *rrs* A514C or *gidB* V77G. The molecular cluster analysis indicated evidence for transmission of resistant strain.

**Conclusions:**

We observed a substantial rate of MDR-TB in the Madang area of PNG associated with mutations in specific genes. A close monitoring of drug resistance is therefore urgently required, particularly in the presence of drug-resistant *M. tuberculosis* transmission. In the absence of phenotypic drug susceptibility testing in PNG, molecular assays for drug resistance monitoring would be of advantage.

## Background

*Mycobacterium tuberculosis* drug resistance is a global concern. In Papua New Guinea (PNG), the estimated tuberculosis (TB) incidence rate is 303/100000 population, with 5% multidrug resistant TB (MDR-TB) among new cases [1]. Culture-based drug susceptibility testing (DST) requires infrastructures often too sophisticated for resource-constrained settings. Detecting resistance-associated mutations is a faster alternative, as shown by Genotype MTBDR*plus* (Hain Life science) [2] or Xpert MTB/RIF (Cepheid) [3]. To monitor drug resistance molecularly, the distribution of drug resistance-conferring mutations in a given setting needs to be known, and such data is currently missing for PNG. We report mutations associated with drug resistance among TB isolates in the Madang area of PNG and provide evidence for transmission of drug-resistant *M. tuberculosis*.

## Results and discussion

The patient characteristics and detailed *M. tuberculosis* genotypes were reported elsewhere [4]. In brief, 60 patients were recruited in the frame of a pilot study in 2005-2007 and 201 in the frame of a treatment cohort study in 2009-2010. History of previous TB treatment was reported in 16.9% (31/201) of the 2009-2010 patients, for whom data was collected. Molecular analyses were performed on the DNA from 173 successfully grown isolates and phenotypic DST was obtained for 172 isolates. From the six previously described *M. tuberculosis* lineages [5], we observed 133/173 (76.9%) Euro-American (Lineage 4), 39/173 (22.5%) East-Asian (Lineage 2, includes Beijing genotype), and 1/173 (0.6%) Indo-Oceanic (Lineage 1).

Overall, 27/172 (15.7%) isolates were resistant to ≥1 drug: 15/172 (8.7%) monoresistant, 3/172 (1.8%) polyresistant and 9/172 (5.2%) MDR. A total of 10/172 (5.8%) strains were Rifampicin (RIF) resistant, 21/172 (12.2%) Isoniazid (INH) resistant (13 low-level [0.1 mg/L], 8 high-level [0.4 mg/L]), 9/172 (5.2%) Streptomycin (STR) resistant, and 4/172 (2.3%) Ethionamide (ETH) resistant.

**Figure 1:**
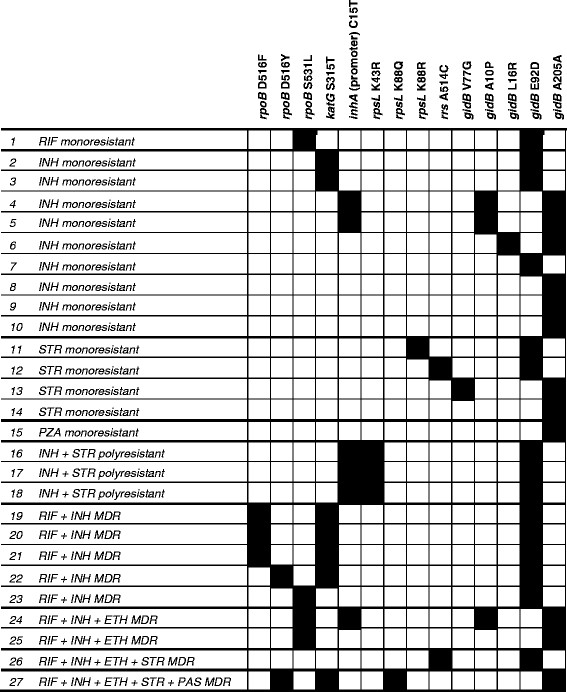
**List of all mutations observed in each of the 27 strains resistant to at least one drug.** The polymorphisms are indicated at codon positions, except for *rrs* gene. *RIF*: Rifampin; *INH*: Isoniazid; *STR*: Streptomycin; *PZA*: Pyrazinamide; *ETH*: Ethionamide; *PAS*: *p*-aminosalicylic acid; *MDR*: Multidrug resistant.

Among resistant isolates, the genes harboring drug resistance associated mutations were sequenced. The observed mutations in *katG*, *inhA* promoter, *ahpC* promoter, *rpoB*, *embB*, *pncA*, *rpsL*, *rrs*, *gidB*, and *gyrA* are listed in Figure 1.

INH resistant isolates harbored mutations in *katG* (codon S315T) or *inhA* promoter (nucleotide C15T). All isolates with *katG* S315T were resistant to 0.4 mg/L INH except one, which was sensitive to this concentration of INH. On the other hand, all isolates with *inhA* promoter mutation were sensitive at this drug concentration (but resistant at 0.1 mg/L), thus confirming the association between *inhA* promoter mutations and low-level INH resistance [6]. Among all 6/9 MDR-TB isolates with either *katG* or *inhA* promoter mutations, all had the *katG* S315T mutation, except one with an *inhA* promoter mutation. This only MDR-TB case with an *inhA* promoter mutation belonged to the four MDR-TB cases, which were additionally ETH resistant. Mutations in *inhA* promoter have been shown to cause INH and ETH cross-resistance and were thereby associated with higher risks of extensively drug resistant TB [7].

Eight INH resistant strains (38.1%) had no *katG* or *inhA* promoter mutation. Only 850 bp of *katG* were sequenced and mutations may therefore have been missed. However, *katG* mutations outside this region are rarer [6,8,9]. Alternatively, these strains might harbor mutation(s) in the >20 other genes reported as potentially associated with INH resistance (genes *iniA* or *x* for example) [8].

We did not observe any *ahpC* promoter mutation, thought to compensate the reduced catalase-peroxidase activity resulting from *katG* mutations [10,11]. Because the INH resistance-conferring mutations observed here, i.e. *katG* S315T and *inhA* promoter C15T, are known to be associated with a low fitness cost [11], they might not require compensation.

All RIF resistant isolates harbored mutations in *rpoB* at codons D516F, D516Y or S531L except one, which did not have any mutation in the 600pb *rpoB* fragment sequenced. DST was repeated for this case, confirming the MDR phenotype. Furthermore, common *rpoBkatG* and *inhA* promoter mutations were excluded by Genotype MTBDR*plus*. Nevertheless, it has been estimated that mutations in the RIF resistance determining region (81-bp region in *rpoB*) account only for 95% of RIF resistance [6] and therefore other mechanisms cannot be excluded. Mutation S531L has been linked to high-level RIF resistance [12], whereas D516Y was associated with low-level resistance [13-15]. Mutation D516F has only been reported in Kazakhstan [16] and may also cause low-level resistance. Low-level RIF resistance has been little considered, but could influence treatment, especially knowing that phenotypic DST outcomes may differ from the actual efficacy of the anti-TB drugs in patients [17].

STR resistant isolates harbored mutations in *rpsL* (codons K43R, K88Q, K88R) and *rrs* (nucleotide A514C), as previously reported [18,19]. One isolate was mutated at codon V77G in *gidB*, a mutation which was not reported before. One STR resistant isolate did not present any mutation in any of these genes.

Mutations in *gidB* have been associated with low-level STR resistance [20,21], but were also reported in sensitive strains [22]. In this study, *gidB* mutations A10P, L16R, E92D, and A205A were observed among strains resistant to other drugs than STR. We further explored *gidB* mutations in whole genomes of 21 pan-susceptible strains representative of the six defined *M. tuberculosis* lineages [23]. Mutation *gidB* V77G, which we observed in one STR resistant isolate from PNG, could not be found in any of the 21 pan-susceptible strains. This mutation could therefore indeed be involved in drug resistance or could be a transitory polymorphism in the population. The mutation A10P observed in one STR sensitive isolate was not found in any of the 21 pan-susceptible genomes. Mutations L16R was observed in genome sequences from Lineage 4 strains (Euro-American lineage) and E92D in Lineage 2 strains (East-Asian lineage). This supports the recent observation that *gidB* L16R occurred in LAM strains (i.e. Lineage 4), whereas *gidB* E92D occurred in Beijing strains [24]. A205A appeared mutated in all strains not belonging to Lineage 4, therefore indicating that this mutation, identified by comparison to H37Rv, is a Lineage 4 mutation. Observations from the 21 pan-susceptible genomes suggest that most *gidB* mutations rather reflect *M. tuberculosis* lineage evolution than drug resistance.

Clusters were defined for strains sharing identical spoligotype and 24 locus mycobacterial interspersed repetitive unit variable number of tandem repeats (MIRU-VNTR) patterns. Among isolates with complete patterns, 72/162 (44.4%) were clustered. Despite potential fitness costs associated with resistance-conferring mutations [25], the proportion of clustered strains was not significantly different among drug-sensitive (60/137, 43.8%) and drug-resistant (12/25, 48.0%) isolates of *M. tuberculosis*.

To distinguish between primary resistance and acquired resistance, clustered isolates sharing identical drug resistance-conferring mutations were considered. Five of the 12 (41.7%) drug-resistant isolates involved in molecular clusters shared their drug resistance-conferring mutations with other isolates in the same cluster, thus strongly suggesting patient-to-patient transmission.

## Conclusions

This study provides so far missing data about drug resistance-conferring mutations in *M. tuberculosis* isolates from Madang in PNG. Monitoring drug resistance is essential to prevent the spread of resistant bacteria, especially in diseases requiring lengthy treatments such as TB. Our data suggests that not all present drug resistance associated mutations may be detected by molecular tests, which mainly focus on a subset of polymorphisms only. However, given the complex implementation of culture-based DST in resource-constrained settings, PNG may be well suited for an accelerated roll-out of molecular drug resistance testing in order to better tackle the emergence and the transmission of drug-resistant *M. tuberculosis* strains.

## Methods

### Study site and patient characteristics

In 2005-2007, a pilot study was conducted in Madang (PNG) at the Modillion Hospital, which is the main point of care in Madang province. In April 2009, a cohort study was initiated in the same hospital and two smaller health centers in close vicinity to Madang town. Patients above 14 years were included if having microscopically confirmed pulmonary TB or other clinical evidence suggesting smear-negative TB. Treatment and follow-up were planed according to the directly observed treatment, short-course (DOTS) program. Demographic and clinical data were available for all patients, except those recruited during the 2005-2007 pilot study.

### Sample processing

Sputum samples were examined by light microscopy after Ziehl-Neelsen staining. Decontamination was conducted according to Petroff’s method [26]. DST was performed by proportion method [27] at the Queensland Mycobacterial Reference Laboratory in Australia using BACTEC™ MGIT™ 960 (Beckton Dickinson, USA) and the following drug concentrations: RIF (1.0 μg/mL), INH (0.1 and 0.4 μg/mL), Ethambutol (5.0 μg/mL), Pyrazinamide (100 μg/mL), Streptomycin (1.0 μg/mL), Amikacin (1.0 μg/mL), Kanamycin (5.0 μg/mL), Ofloxacin (2.0 μg/mL), Capreomycin (2.5 μg/mL), ETH (5.0 μg/mL), *p*-Aminosalicylic acid (4.0 μg/mL), and Cycloserine (50.0 μg/mL). Isolates resistant to one drug were categorized as monoresistant, isolates resistant to at least INH and RIF were categorized as MDR and isolates resistant to at least one drug but not MDR were considered polyresistant. DNA was extracted from cultures using Instigate Matrix (Bio-Rad, USA) and sent to the Swiss Tropical and Public Health Institute for molecular analyses.

### Strain genotyping

Spoligotyping and 24 locus MIRU-VNTR were used to define strain clusters as previously described [28,29]. The online MIRU-VNTR*plus* platform was used for cluster identification ( http://www.miru-vntrplus.org[30]). Clusters were defined for strains sharing identical spoligotype and 24 locus MIRU-VNTR patterns. Strains were assigned to one of the six previously described lineages by real-time PCR identification of specific single nucleotide polymorphisms (SNPs) [5,31-33].

### Drug resistance mutations

The following genes (or gene regions) were sequenced to capture drug resistance conferring SNPs: *rpoBkatGinhA* promoter, *ahpC* promoter, *embBpncArpsLrrsgidB*, and *gyrA* (see Additional file 1: Table S1 for primers and PCR conditions). Sequencing was performed by Macrogen (The Netherlands). Observed mutations were compared to the online Tuberculosis Drug Resistance Mutation Database (TBDream, http://www.tbdreamdb.com[8]).

## Ethical approval

The PNG Institute for Medical Research Review Board, and the PNG National Medical Research Advisory Council’s Ethics Committee approved the study protocol. The Ethikkommission beider Basel in Switzerland was informed about the study. Written informed consent was obtained from all patients enrolled in the study.

## Competing interests

The authors declare that they have no competing interests.

## Authors’ contributions

MB carried out the molecular analyses, the data analyses and drafted the manuscript. PH conducted the patient recruitment and follow-up. SL participated to the study design. MC conducted the whole genome analyses. SN conducted the MIRU-VNTR analyses. RC conducted the phenotypic DST. CC participated in the phenotypic DST and helped to draft the manuscript. SB advised the molecular work and helped to draft the manuscript. PS contributed to the study set up. SP conceived the study design. SG participated in the design of the study, coordinated the molecular work and helped to draft the manuscript. Hans-Peter Beck participated in the design of the study, coordinated the molecular work and helped to draft the manuscript. All authors read and approved the final manuscript.

## Authors’ information

Co-senior author: Sebastien Gagneux and Hans-Peter Beck.

## Supplementary Material

Additional file 1 Table 1.Primers and PCR conditions. Click here for file
