# Supplementary material for: Drug resistance-conferring mutations in Mycobacterium tuberculosis from Madang, Papua New Guinea
Source: BMC Microbiol. 2012 Sep 4;12:191. doi: 10.1186/1471-2180-12-191 (PMC3478209; doi:10.1186/1471-2180-12-191)
Supplement: Additional file 1 — Table 1.Primers and PCR conditions. [file 1471-2180-12-191-S1.doc]

## Supplementary table 1:

Primers and PCR conditions.

| **Gene** | | **Direction** | **Primer sequence (5’-3’)** | **PCR conditions** | **Product length (bp)** | **Reference** |
| --- | --- | --- | --- | --- | --- | --- |
| *rpoB* | Rv0667 | Forward | TCGGCGAGCTGATCCAAAACCA | Tm: 62°C, Ex: 45, Cy: 35 | 601 | This study |
| *rpoB* | Rv0667 | Reverse | ACGTCCATGTAGTCCACCTCAG |  |  | This study |
| *inhA* (promoter) | Rv1484 | Forward | GGCACGTACACGTCTTTATGTA | Tm: 60°C, Ex: 30, Cy: 35 | 478 | [1] |
| *inhA* (promoter) | Rv1484 | Reverse | GGTGCTCTTCTACCGCCGTGAA |  |  | [1] |
| *katG* | Rv1908c | Forward | CCAGCGGCCCAAGGTATC | Tm: 64°C, Ex: 60, Cy: 35 | 850 | This study |
| *katG* | Rv1908c | Reverse | GCTGTGGCCGGTCAAGAAGAAGTA |  |  | This study |
| *ahpC* (promoter) | Rv2428 | Forward | ACCACTGCTTTGCCGCCACC | Tm: 64°C, Ex: 30, Cy: 35 | 236 | [2] |
| *ahpC* (promoter) | Rv2428 | Reverse | CCGATGAGAGCGGTGAGCTG |  |  | [2] |
| *embB* | Rv3795 | Forward | CGGCATGCGCCGGCTGATTC | Tm: 65°C, Ex: 30, Cy: 35 | 260 | [3] |
| *embB* | Rv3795 | Reverse | TCCACAGACTGGCGTCGCTG |  |  | [3] |
| *gyrA* | Rv0006 | Forward | CAGCTACATCGACTATGCG | Tm: 58°C, Ex: 30, Cy: 35 | 320 | [4] |
| *gyrA* | Rv0006 | Reverse | GGCTTCGGTGTACCTCATC |  |  | Adapted from [4] |
| *gidB* | Rv3919c | Forward | CGAGAGCGGAGAATGTTTCAC | Tm: 60°C, Ex: 60, Cy: 35 | 793 | This study |
| *gidB* | Rv3919c | Reverse | CTGGCCCGACCTTACGAGC |  |  | This study |
| *rpsL* | Rv0682 | Forward | CGTGAAAGCGCCCAAGATAG | Tm: 62°C, Ex: 30, Cy: 35 | 333 | This study |
| *rpsL* | Rv0682 | Reverse | GAACCGCGGATGATCTTGTAG |  |  | Adapted from [5] |
| *rrs* | MTB000033 | Forward | GATGACGGCCTTCGGGTTGT | Tm: 60°C, Ex: 30, Cy: 35 | 238 | [5] |
| *rrs* | MTB000033 | Reverse | TCTAGTCTGCCCGTATCGCC |  |  | [5] |
| *rrs* | MTB000033 | Forward | GTAGTCCACGCCGTAAACGG | Tm: 60°C, Ex: 30, Cy: 35 | 245 | [5] |
| *rrs* | MTB000033 | Reverse | CACACAGGCCACAAGGGAAC |  |  | Adapted from [5] |
| *rrs* | MTB000033 | Forward | CGTTCCCTTGTGGCCTGTG | Tm: 62°C, Ex: 45, Cy: 35 | 547 | This study |
| *rrs* | MTB000033 | Reverse | GGCGTTTTCGTGGTGCTCC |  |  | Adapted from [5] |
| *pncA* | Rv2043c | Forward | GGCTGCCGCGTCGGTAGG | Tm: 62°C, Ex: 45, Cy: 35 | 652 | This study |
| *pncA* | Rv2043c | Reverse | GCCGCCAACAGTTCATCCC |  |  | This study |

All PCR were performed using FastStart Taq DNA Polymerase and corresponding buffers (Roche Diagnostics, Switzerland). Tm: Annealing temperature, Ex: Extension time (seconds), Cy: number of cycles.

References to Supplementary table 1:

[1] Gagneux S *et al*. Impact of bacterial genetics on the transmission of isoniazid-resistant *Mycobacterium tuberculosis*, PLoS Pathogens, 2006

[2] Homolka S *et al*. Unequal distribution of resistance-conferring mutations among *Mycobacterium tuberculosis* and *Mycobacterium africanum* strains from Ghana, Int J Med Microbiol, 2010

[3] Victor TC *et al.* Detection of mutations in drug resistance genes of *Mycobacterium tuberculosis* by a dot-blot hybridization strategy, Tuber Lung Dis, 1999

[4] Feuerriegel S *et al.* Sequence analyses of just four genes to detect extensively drug-resistant *Mycobacterium tuberculosis* strains in multidrug-resistant tuberculosis patients undergoing treatment, Antimicrob Agents Chemother, 2009

[5] Brossier F *et al.* Detection by GenoType MTBDRsl test of complex mechanisms of resistance to second-line drugs and ethambutol in multidrug-resistant *Mycobacterium tuberculosis* complex isolates, J Clin Microbiol, 2010
